# Rate and pattern of unintentional injuries among 9-12 grades schoolchildren in Yemen and their associated factors

**DOI:** 10.5249/jivr.v10i2.966

**Published:** 2018-07

**Authors:** Ahmed Alshahethi, Abdulwahed Al Serouri, Yousef S Khader

**Affiliations:** ^*a*^Yemen-Field Epidemiology Training Program, Ministry of Health Surveillance Building, Mazda Street, Sana'a, Yemen.; ^*b*^ Department of Community Medicine, Public Health and Family Medicine, Jordan University of Science & Technology.

**Keywords:** Unintentional injuries, Schoolchildren, Associated factors, Field Epidemiology, Training program, Yemen

## Abstract

**Background:**

The burden and pattern of unintentional child injuries in Yemen are not yet studied. This study aimed to determine the rate of unintentional injuries and their associated factors and describe the pattern of these injuries among schoolchildren in Sana'a city, Yemen. Methods: A cross-sectional school-based study was conducted among students in grades 9 –12 in Sana’a Capital City. A total of 10 schools were selected using multistage sampling technique. A self-administered questionnaire was used to collect the data.

**Methods:**

A cross-sectional school-based study was conducted among students in grades 9 –12 in Sana’a Capital City. A total of 10 schools were selected using multistage sampling technique. A self-administered questionnaire was used to collect the data.

**Results:**

A total of 1140 students (558 girls and 582 boys) participated in the study. Of all students, 550 (48.2%) students reported unintentional injuries during the last 12-months. In the multivariate analysis, boys were more likely to be injured compared to girls (OR = 1.6) and being a child of divorced or widowed parents was significantly associated with increased odds of injury (OR = 1.7). Age of the household head ≤ 45 years was associated with deceased odds of injuries (OR = 0.76). Fall was the leading cause of injury. More than half of girls (58.9%) and 30.9% of boys were injured at home. About two thirds (64.9%) of injuries affected the lower or upper extremities. One quarter of students (24.5%) received care for their injuries in outpatient clinics and 15.3% were hospitalized because of the injury. About 26.0% of injured students missed schools for one week or more. The vast majority of students (98.4%) recovered the injury while 1.6% of injuries resulted in disability.

**Conclusions:**

Schoolchildren in Yemen had a high rate of unintentional injuries being higher in boys and in children of divorced or widowed parents. These injuries should be recognized as a public health problem in Yemen and should be included in the Ministry of Education and Ministry of Health agenda. The reported injury mechanisms and activities posing injury risks should have implications for future interventions and awareness programs.

## Introduction

Childhood injury is a growing public health problem^1^ with road traffic, drowning, burns, falls, and poisonings being the leading causes of injuries.^2-3^ For every injured child who dies, several thousand more survive with varying degrees of disability.^4^ Previous studies have estimated the annual injury rates from 5% to 50% among students and indicated that school-age children are nine times more likely to sustain an accidental injury.^5-7^ In addition to the medical consequences and significant health loss, concomitant educational failure could result due to extended absence from school. Such high incidence rates and consequences call for preventive measures to combat accidents among schoolchildren.

Identifying children at risk of injury is very important to strengthen strategies and develop necessary interventions to prevent unintentional injuries among children. Childhood injuries result from an unsafe interaction between the child and the cause of the injury. Many factors had been identified to affect the risk of injury among children including individual,^3-8^ family ^9^ and community level factors. ^10^ Other factors related to the surrounding physical and social environment play an important role on the occurrence of injuries. However the actual contributions of these factors to the burden of children’s injuries in the general population remain unclear. ^10^

The burden of unintentional child injuries in the Eastern Mediterranean Region (EMR) is one of the highest in the world. The EMR endures about 12% of all unintentional injury deaths of the world in those under 20 years of age.^2^ In Yemen, child injuries are largely absent from child survival initiatives and from the Ministry of Public Health and Population (MoPHP) and Ministry of Education (MoE) agenda and their burden and patterns are not yet studied. Understanding the patterns and determinants of injuries is a prerequisite before launching any prevention programs or designing any awareness rising activities. Therefore, this study aimed to determine the rate of unintentional injuries and their associated factors and describe the pattern of these injuries among schoolchildren in Sana'a city, Yemen.

## Methods 

**Study design **

A cross-sectional school-based study was conducted among students in grades 9 –12 in Sana’a Capital City. A total of 10 schools in ten districts of the Sana'a city were selected using multistage random sampling technique based on the school geographical location and the total number of classes. A sample of 1200 students was randomly selected from 40 classes of 9-12 grades in 10 schools at Sana'a Capital City, Yemen. Ethical approval was obtained from the National Ethical Committee at the Ministry of Public Health and Population, Yemen.

**Data collection**

A permission to conduct the study in the school premises was obtained from Sana'a city Education Bureau. The interviewer read the Arabic consent statement and explained the study objectives and procedures to the respondents. All participants had provided the consent to participate according to the Declaration of Helsinki. 

A self-administered questionnaire was used to collect the data. The questionnaire included questions about socio-demographic characteristics of students (age and gender) and their families (number of children in the family, family income, current marital status of parents, and family head education). Students were asked to report any injury event in the past 12 months. An injury was defined as an injury meeting at least one of the following criteria: (1) an injury for which the student received medical treatment at the school nurse's office, or received medical care from a doctor at a hospital or a private medical facility, (2) an injury for which the student received first aid from his/her schoolmates, teachers, or parents, or (3) an injury that was not treated but caused the student to miss a half day or more of school or regular activities. Data about the last injury in the last 12 months were also collected and included date and time of the injury, cause of the injury, activity when injured, location of the injury, person(s) who caused the injury, body part injured, school days or activities missed because of the injury, medical advice or treatment received after the injury and injury outcomes. For analyses of injuries by cause, ICD-10 was used to group them into categories (e.g. traffic, fall, cut, burn, etc) with block T90 of code range (S00-T98). The aim of this study was to determine the rate of unintentional injuries and their associated factors, therefore, we excluded intentional injuries (e.g. child abuse, suicide, and homicide) as studying these injuries would face cultural and legal constraints that may require using a different design. 

The questionnaire was pilot tested on 50 students. The findings of pilot testing were used to finalize the study questionnaire. Data collectors received formal training by researchers from Yemen-Field Epidemiology Training Program (FETP). The time needed to fill the questionnaire was 15-20 minutes. 

**Statistical Analysis**

Data were described and analyzed using SPSS IBM, version 20. Data were described using percentages, ratios, and frequency tables. The 12-months prevalence rate of unintentional injury among 9 to 12 grades school students were compared according to students’ characteristics using Chi-square test. Multivariate analysis using binary logistic regression was used to determine factors associated with injury. Crude and adjusted odds ratios and their 95% confidence intervals (CIs) were reported. Significant variables were selected using backward stepwise selection method.^11^ A p-value <0.05 was considered statistically significant. 

## Results

**Participants’ characteristics**

A total of 1140 students (558 girls and 582 boys) responded and filled the questionnaire with a response rate of 95%. About two thirds (60.7%) of students were from public schools and 39.3% were from private schools. Their age ranged from 15 to 18, with a mean (SD) of 16.5 (1.1) year. 

**Prevalence rate of injury and its associated factors**

Of all students, 550 (48.2%) students reported unintentional injuries during the last 12-months. Among those who reported that they were being injured in the last 12-months, 58.2% had more than one injury. Table 1 shows the 12-months period prevalence rates of injury according to the participants’ characteristics. 

**Table 1:** The 12-months prevalence rate of unintentional injury among 9 to 12 grades school students, Yemen.

	Not injured		Injured		Total	P-value
	n	%	n	%		
**Gender**						**<0.001**
Female	322	57.7	236	42.3	558	
Male	268	46.0	314	54.0	582	
**Age (year)**						**0.215**
15	142	50.7	138	49.3	280	
16	146	51.0	140	49.0	286	
17	142	48.5	151	51.5	293	
18	160	56.9	121	43.1	281	
**Grade**						**0.164**
9	142	50.4	140	49.6	282	
10	148	52.1	136	47.9	284	
11	140	47.8	153	52.2	293	
12	160	56.9	121	43.1	281	
**School type**						**0.150**
Public	370	53.5	322	46.5	692	
Private	220	49.1	228	50.9	448	
**Number of children in the family**						**0.087**
1-2	112	49.1	116	50.9	228	
3-4	154	47.1	173	52.9	327	
5-6	178	55.3	144	44.7	322	
>6	146	55.5	117	44.5	263	
**Age of the household head **						**0.168**
≤45	358	53.5	311	46.5	669	
>45	229	49.4	235	50.6	464	
**Education level of the household head**						**0.130**
Illiterate	76	51.7	71	48.3	147	
High school or less	346	54.1	293	45.9	639	
>High school	168	47.5	186	52.5	354	
**Employment status of household head**						**0.448**
Not employed	89	49.2	92	50.8	181	
Employed	501	52.2	458	47.8	959	
**Marital Status**						**0.011**
Married	549	52.9	488	47.1	1,037	
Divorced/Widow	41	39.8	62	60.2	103	

The prevalence rate of injury was significantly higher among boys compared to that among girls (54.0% vs. 42.3%; p-value<0.001). Children whose parents were divorced or widowed had higher prevalence of injury rate compared to those children whose parents were married at the time of injury (60.2% vs. 47.1%; p-value = 0.011). The prevalence rate of injury did not differ significantly according to other studied characteristics. Girls of divorced/widowed parents had increased odds of injury (OR = 2.5, 95% confidence interval (CI): 1.4, 4.4; p-value = 0.002) compared to boys of divorced/widowed parents (Table 2).

**Table 2:** The 12-months prevalence rate of unintentional injury among 9 to 12 grades school students stratified by student's gender and marital status of parents.

Students' gender	Parents' marital status	Injured n (%)	No Injured n (%)	ORa	95% CI^b^	P-value
**Male**	**Divorced/Widowed**	29 (58.0)	21 (42.0)	1.19	0.64, 2.23	0.560
	**Married**	286 (53.7)	247 (46.3)	Ref		
**Female**	**Divorced/Widowed**	33 (54.0)	20 (46.0)	2.47	1.33, 4.60	0.002
	**Married**	203 (40.2)	302 (59.8)	Ref		

a: OR -Odds Ratio, b: 95% CI- Confidence interval

In the multivariate logistic regression analysis(Table 3), the only variables that were significantly associated with injury were child’s gender, parents’ marital status and age of the household head. Boys were more likely to be injured compared to girls (OR = 1.60, 95% confidence interval (CI): 1.21, 2.04; p-value<0.001). Being a child of divorced or widowed parents was significantly associated with increased odds of injury (OR = 1.9, 95% confidence interval (CI): 1.2, 2.9; p-value = 0.004). Age of the household head ≤ 45 years was associated with deceased odds of injuries (OR = 0.76, 95% confidence interval (CI): 0.59, 0.98; p-value = 0.035).

**Table 3:** Multivariate analysis of factors associated with unintentional injury among 9 to 12 grades school students in Sana'a City, Yemen, 2012.

Variable	Crude OR	Adjusted OR	95% confidence interval (adjusted OR)	P-Value
**Gender**				
Male vs. Female	1.6	1.57	1.21, 2.04	0.001
**Age of the household head (year)**				
≤ 45 vs. > 45	0.84	0.76	0.59, 0.98	0.035
**Marital status**				
Divorced/Widowed vs. Married	1.7	1.88	1.23, 2.88	0.004

**Injury characteristics and outcomes**

Table 4 shows the injury characteristics and outcomes according to gender. For both boys and girls, fall was the leading cause of injury (51.1% among boys and 43.3% among girls). More than half of girls (58.9%) and 30.9% of boys were injured at home. Boys were more likely to be injured at schools compared to girls (32.1% vs. 23.3%). About two thirds (64.9%) of injuries affected the lower or upper extremities. One quarter of students (24.5%) received care for their injuries in outpatient clinics and 15.3% were hospitalized because of the injury. No medical care was received by 24.0% of injured students. About 26.0% of injured students missed schools for one week or more. The vast majority of students were recovered (98.4%) while 1.6% of injuries resulted in disability. 

**Table 4:** The characteristics of unintentional injuries among 9 to 12 grade school students according to gender.

	Male(n=314) n (%)	Female (n=236) n (%)	Total (N=550) n (%)
**Cause of injury**			
Transportation	42 (13.4)	28 (11.9)	70 (13.0)
Burn	11 (3.5)	34 (14.4)	45 (8.0)
Falls	161 (51.1)	102 (43.4)	263 (48.0)
Struck	38 (12.4)	27 (11.5)	65 (12.0)
Animal/Insect bite	10 (3.2)	7 (3.0)	17 (3.0)
Cut/Pierce	15 (4.8)	10 (4.2)	25 (5.0)
Choking	6 (1.9)	6 (2.5)	12 (2.0)
Poisoning	7 (2.2)	10 (4.3)	17 (3.0)
Drowning	3 (1.0)	2 (0.8)	5 (1.0)
Open wound	18 (5.7)	8 (3.4)	26 (5.0)
Other (e.g. gunshot, pin)	3 (1.0)	1 (0.4)	4 (0.7)
**Location at injury **			
Home	97 (30.9)	139 (58.9)	236 (42.9)
School	101(32.1)	55 (23.3)	156 (28.3)
From/To School	81 (25.8)	35 (14.8)	116 (21.1)
Street	23 (7.3)	7 (1.3)	30 (5.5)
Others	12 (3.8)	0 (0.00)	12 (2.2)
**Activity**			
Riding bicycle/Car	50 (15.9)	6 (2.5)	56 (18.2)
Home chores	17 (5.4)	58 (24.6)	75 (13.6)
Sport	74 (23.6)	13 (5.5)	87 (15.8)
Leisure	45 (14.3)	34 (14.4)	79 (14.4)
Walking	61 (19.4)	59 (25.0)	120 (21.8)
Descending stair	27 (8.6)	31 (13.1)	58 (10.5)
Climbing stair	10 (3.2)	4 (1.7)	14 (2.5)
During eating/drinking	13 (4.1)	22 (9.3)	35 (6.4)
Bathing	8 (2.6)	7 (2.9)	15 (2.7)
Other (e.g. fight, travel)	9 (2.9)	2 (0.95)	11 (2.0)
**Person(s) who caused injury**			
Self	171 (54.5)	162 (68.7)	333 (61.0)
Schoolmate	102 (32.5)	27 (11.4)	129 (23.5)
Other (e.g. teacher, relative)	41 (13.1)	47 (19.9)	88 (16.0)
**Body part injured **			
Head/Face	35 (11.2)	21 (8.9)	56 (10.2)
Eyes	2 (0.64)	4 (1.7)	6 (1.1)
Teeth	5 (1.6)	4 (1.7)	9 (2.0)
Upper Limbs	81 (25.8)	75 (31.8)	156 (28.4)
Lower Limbs	126 (40.1)	75 (31.8)	201 (36.5)
Back	20 (6.4)	11 (4.7)	31 (5.6)
Chest	11 (3.5)	7 (2.9)	18 (3.3)
Multiple	34 (10.8)	39 (16.5)	73 (13.3)
**Care received after injury **			
By teacher	10 (3.2)	18 (7.6)	28 (5.1)
By parents	62 (19.8)	80 (33.9)	142 (25.8)
By school staff	15 (4.8)	14 (5.9)	29 (5.3)
Outpatient clinics	87 (27.7)	48 (20.3)	135 (24.5)
Hospitalization	51 (16.2)	33 (14.1)	84 (15.3)
No care received	89 (28.3)	43 (18.2)	132 (24.0)
**School days missed after injury**			
1	59 (18.8)	45 (19.7)	104 (18.9)
2-3	58 (18.5)	75 (31.8)	133 (24.2)
4-6	38 (12.1)	17 (7.2)	55 (10.0)
≥7	101(32.2)	42 (17.8)	143 (26.0)
No absent	58 (18.5)	57 (24.2)	115 (20.9)
**Injury outcome**			
Recovery	307 (97.8)	234 (99.2)	541 (98.4)
Disability	7 (2.2)	2 (0.85)	9 (1.6)

## Discussion

Childhood unintentional injuries are prevalent in low and middle income countries and they are largely preventable cause of death and disability. Existing studies indicate that the rates of unintentional injuries are significantly higher in low middle income countries compared to high income countries. ^12^ Results from previous studies showed a high variability in the injury rates. The comparison of the findings of these studies is challenging because they had different study periods, injury definitions and data collection methods. 

About half of students had unintentional injuries during the last 12-months. This rate was among the highest rates reported in studies with similar designs.^13,14^ In this study, males had significantly higher overall injury rates than females, which is consistent with other studies. ^15^ This might be explained by the fact that boys take more risk than girls and have higher activity levels. However, the gender difference in injury rate depends on the type of injury. In the present study, girls had higher prevalence of fire-related injuries which is consistent with the findings of other studies in poor countries^4,16^ because they are involved in cooking in open and unsafe kitchens.^4^

Marital status of the parents was shown to be associated with the child injuries; a finding that has been reported in previous studies.^17,18^ Dawson ^17^ had indicated that children who lived in divorced families had greater risk for injury. This finding might be explained by that children who live in divorced families are less likely to receive attention from their parents. Age of the household head ≤ 45 years was associated with deceased odds of injuries. This finding might be explained by that younger parents have fewer children compared to older parents and this might provide children with higher opportunity of attention and supervision. However, this finding is not in agreement with the findings of other studies.^19^

The rate of injury was not associated with child's age in this study. This finding contradicts the findings of other studies^20,21^ that reported age-related injury rates and patterns. The lack of the association between injury and child's age might be explained by the small variation in age of children that ranged from 15 to 18 years. This study did not support the association between other socioeconomic status and injury that has been demonstrated in other studies. ^22,23^ It is worth mentioning that monthly income was not reported by 36.4% of children. 

Our results regarding the leading causes of injury, activity when injured, location of injury, and body part injured among school children were consistent with the findings from other studies.^24^ Fall was the leading cause of injury among boys and girls. Less than half of girls and about two thirds of boys are injured outside homes, either in school or street especially during sport activities. Therefore, physical education teachers and coaches at schools need to be aware of the high risk of injury associated with sports activities and should design age-appropriate activities for students to reduce the risk of injury.

Parents in cooperation with safety authorities need to focus and implement appropriate environmental injury prevention strategies, particularly those occurring among children at home (e.g. use of stair gates, smoke alarm and a four-sided fence with a locked gate around residential swimming pools) and at the way to/from school such as appropriate restrains in car (i.e., booster seats, seat belts), bicycle helmets and improved pedestrian safety. Furthermore, seat belts and child restraints laws should be enforced. 

One of the limitations of this study is the recall bias as some students may not remember an injury or its details that occurred during the past year. Another limitation is that the findings of this study might not reflect the injury risk and patterns among the entire school population of Yemen. 

In conclusion, schoolchildren in Yemen had a high rate of unintentional injuries during the last 12-months being higher in boys and children of divorced or widowed parents. Unintentional injury among schoolchildren in Yemen should be recognized as a public health problem in Yemen and should be included in the Ministry of Education and Ministry of Health agenda. The reported injury mechanisms and activities posing injury risks should have implications for future interventions and awareness programs and should guide school environment improvement. The schools and other authorities that organize sports programs and recreational activities need to emphasize the importance of safety equipment for the particular sport as well as appropriate physical conditioning for that sport.

**Acknowledgements**

Authors would like to acknowledge the Training Programs in Epidemiology & Public Health Interventions Network (TEPHINET) and Yemen Field Epidemiology Training Program for their technical support.
